# Muscleblind1, but Not Dmpk or Six5, Contributes to a Complex Phenotype of Muscular and Motivational Deficits in Mouse Models of Myotonic Dystrophy

**DOI:** 10.1371/journal.pone.0009857

**Published:** 2010-03-25

**Authors:** Anna Matynia, Carina Hoi Ng, Warunee Dansithong, Andy Chiang, Alcino J. Silva, Sita Reddy

**Affiliations:** 1 Departments of Neurobiology, Psychiatry & Biobehavioral Sciences, Psychology and the Brain Research Institute, Gonda Neuroscience and Genetics Center, University of California Los Angeles, Los Angeles, California, United States of America; 2 Institute for Genetic Medicine, University of Southern California, Los Angeles, California, United States of America; Tokyo Medical and Dental University, Japan

## Abstract

Assessment of molecular defects that underlie cognitive deficits observed in mendelian disorders provides a unique opportunity to identify key regulators of human cognition. Congenital Myotonic Dystrophy 1 (cDM1), a multi-system disorder is characterized by both cognitive deficits and a spectrum of behavioral abnormalities, which include visuo-spatial memory deficits, anxiety and apathy. Decreased levels of DMPK (Dystrophia Myotonica-protein kinase), SIX5, a transcription factor or MBNL1 (Muscleblind-like 1), an RNA splice regulator have been demonstrated to contribute to distinct features of cDM1. Mouse strains in which either *Dmpk*, *Six5* or *Mbnl1* are inactivated were therefore studied to determine the relative contribution of each gene to these cognitive functions. The open field and elevated plus maze tasks were used to examine anxiety, sucrose consumption was used to assess motivation, whereas the water maze and context fear conditioning were used to examine spatial learning and memory. Cognitive and behavioral abnormalities were observed only in *Mbnl1* deficient mice, which demonstrate behavior consistent with motivational deficits in the Morris water maze, a complex visuo-spatial task and in the sucrose consumption test for anhedonia. All three models of cDM1 exhibit normal spatial learning and memory. These data identify MBNL1 as a potential regulator of emotional state with decreased MBNL1 levels underlying the motivational deficits observed in cDM1.

## Introduction

Myotonic dystrophy (DM1) is a multi-system disorder characterized by muscle weakness, skeletal myotonia, cardiac conduction defects, ocular cataracts and cognitive and behavioral abnormalities [1]. DM1 is caused by a CTG repeat expansion found in the 3′UTR of the *DMPK* gene and located immediately 5′ of the *SIX5* gene on chromosome 19 [2], [3], [4], [5]. Repeat expansion results in three distinct molecular defects that increase in severity as a function of repeat tract length: first, as consequence of the aberrant sequestration of the mutant *DMPK* RNA within the nucleus, DMPK levels are decreased in DM1 cells [6], [7]. Second, CTG repeat expansion results in the transcriptional down-regulation of the linked *SIX5* allele leading to diminished levels of SIX5 [8], [9]. Third, expression of expanded CUG repeat sequences have been shown to dysregulate RNA splicing due to the inactivation of the alternative splice regulator, MBNL1, by mechanisms that have yet to be fully understood [10], [11], [12], [13], [14], [15]. The pleiotropy of the DM1 phenotypes are thought to result from a combination of these three effects, with the expression of CUG repeats playing a prominent role in the development of major aspects of the disease.

Patients with the myotonic dystrophy type 1 exhibit cognitive and behavioral abnormalities including mental retardation, visuo-spatial memory deficits, apathy, anxiety, hypersomnolence, autism, depression and attention deficit hyperactivity disorders [16], [17], [18], [19], [20]. Patient assessment suggests that apathy cannot be accounted for by depression or muscle weakness and reflects a CNS involvement [17]. It is also of interest to note that visuo-spatial reasoning and memory appears to be specifically compromised in DM1 patients [16]. The severity of psychiatric illness in DM1 is roughly proportional both to the CTG tract length and age of disease onset with cognitive and behavioral deficits showing the greatest prevalance and severity in the congenital form of the disorder [21].

To investigate the contribution of these genes to cognitive symptoms observed in DM1 patients, we studied the effect of deletion of *Six5*, *Dmpk* and *Mbnl1* on three separate behavioral tasks designed to test learning and memory, anxiety and motivation. These three mouse models of DM1 show various physical phenotypes pertaining to myotonic dystrophy. *Dmpk^−/−^* mice display muscle weakness and heart disease [22], [23], *Six5^−/−^* mice display cataracts and heart disease [24], [25] and *Mbnl1^−/−^* mice display skeletal myotonia, muscle weakness, cataracts and possibly heart disease [15]. Therefore, we carefully chose tasks that require varying degrees of muscular strength (i.e. low demands in fear conditioning, higher demands in the water maze) and performed control tasks when possible (i.e. visible watermaze to control for the hidden water maze) in order to dissociate muscular/physical impairments from higher level brain functional deficits. Our results demonstrate that neither Six5 nor DmpK affect cognitive function but that *Mbnl1^−/−^* mice have deficits consistent with decreased motivation.

## Results

### *Mbnl1^−/−^* mice exhibit contradictory anxiety behaviors

**Figure 1:**
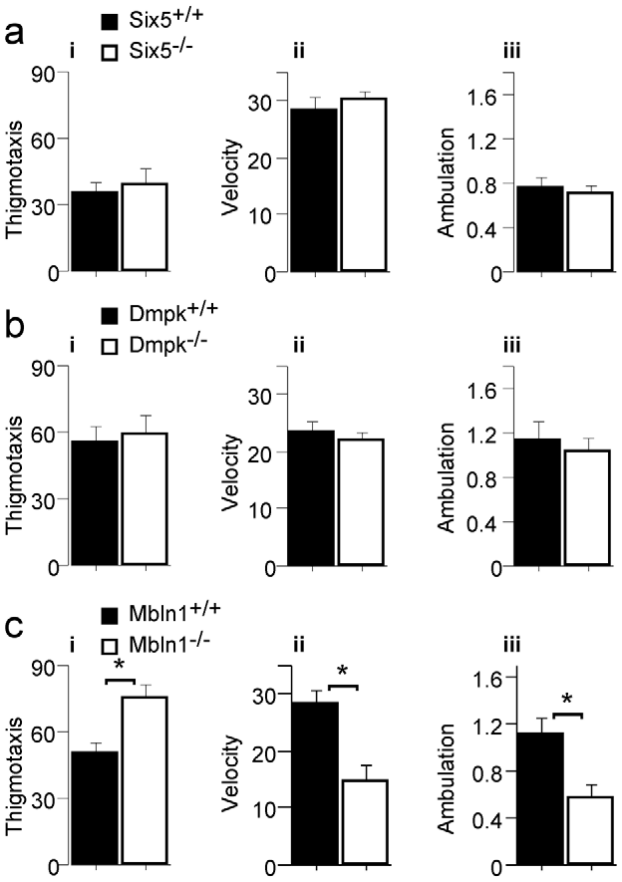
Inactivation of *Six5* or *Dmpk* does not affect anxiety or general motor activity in the Open Field task. Anxiety, as indicated by thigmotaxis, is shown as % time hugging wall (panels i). Activity is shown as velocity (cm/s, panels ii) and ambulation (m traveled, panels iii). A) *Six5^−/−^* (white, n = 7) mutant mice display normal thigmotaxis, velocity and distance travelled compared to their *Six5^+/+^* wild-type littermate controls, (black, n = 6) (panel i, ii, and iii respectively). B) *Dmpk^−/−^* mutant mice (white, n = 9) display normal thigmotaxis, velocity and distance travelled compared to their *Dmpk^+/+^* wild-type littermate controls, (black, n = 9) (panel i, ii and iii, respectively). C) *Mbnl1^−/−^* (white, n = 23) mutant mice display increased thigmotaxis (F(1, 04) = 10.3, p = 0.0026), decreased velocity (F(1,40) = 14.0, p = 0.0006) and decreased distance travelled (F(1,40) = 8.10, p = 0.0070) compared to their *Mbnl1^+/+^* wild type littermate controls (black, n = 19) (panel i, ii and iii, respectively).

We began by assessing anxiety-related behaviors (thigmotaxis or wall-hugging) and general activity using the Open Field task. In this task, mice are placed in an open arena and allowed to explore. No differences in Open Field thigmotaxis (wall-hugging, Figure 1i), ambulation (total cm traveled, Figure 1ii) or average velocity (cm/s, Figure 1iii) were detected in the *Six5^−/−^* and *Dmpk^−/−^* mutant mice compared to their wild-type littermate controls (Figure 1A, and B, respectively). However, *Mbnl1^−/−^* mice showed increased thigmotaxis, and decreased ambulation and average velocity (Figure 1C). These results show that *Mbnl1^−/−^* mice have decreased overall activity experience. Increased thigmotaxis exhibited by the *Mbnl1^−/−^* mice could be due to muscle weakness, increased anxiety or decreased motivation to explore.

Since *Mbnl1^−/−^* mice showed increased thigmotaxis, but the cause of this anxiety-related behavior is unclear, we further tested these mutants in the elevated plus maze, another anxiety test. Mice are placed on a 4-arm maze in which two arms are sheltered and the other two arms are open. This configuration presents a “safe” environment in the sheltered, darker arms and a more “dangerous” environment in the two open, lighted arms. Similar to the open field task, mice that are anxious will spend more time in the “safe”, closed arms.

**Figure 2:**
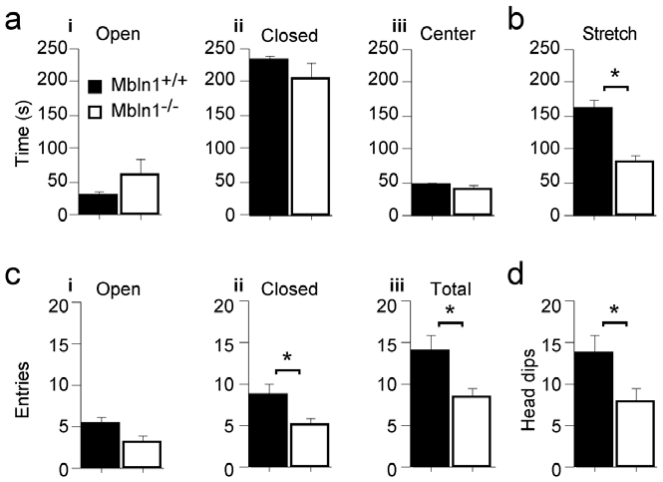
Inactivation of *Mbnl1* does not alter anxiety in the Elevate Plus Maze. A) *Mbnl1^−/−^* (white, n = 14) mutant mice spent the same amount of time in the open (F(1,26) = 0.76, p = 0.39) and closed arms (F(1,26) = 0.40, p = 0.53), and the center (F(1,26) = 0.52, p = 0.48) of the elevated plus maze compared to their *Mbnl1^+/+^* wild type littermate controls (black, n = 14) (panel i, ii and iii, respectively), although the *Mbnl1^−/−^* mice show trends towards decreased anxiety by spending more time in the open arms. B) *Mbnl1^−/−^* mutant mice spent the significantly less time in a stretch posture, indicative of decreased anxiety (F(1,26) = 23, p<0.0001) C) *Mbnl1^−/−^* (white, n = 14) mutant mice display decreased overall activity, specifically decreased total entries into arms (F(1,26) = 8.4, p = 0.0076) which is due to decreased entries into the closed arms (F(1,26) = 9.4, p = 0.005) and not into open arms (F(1,26) = 3.6, p = 0.069) and compared to their *Mbnl1^+/+^* wild type littermate controls (black, n = 14) (panel i, ii and iii, respectively). D) *Mbnl1^−/−^* (white, n = 14) mutant mice display decreased numbers of head dips (F(1,26) = 5.1, p = 0.032) compared to their *Mbnl1^+/+^* wild type littermate controls (black, n = 14), incidcative of increased anxiety, in contrast to B).

The *Mbnl1^−/−^* mice generally show normal anxiety levels in this test. They did not show a preference for the closed arms, spending the same amount of time in the open and closed arms as well as the center of the maze (Figure 2A, I, ii and iii, respectively). Although not statistically significant, the *Mbnl1^−/−^* mice spend slightly more time in the open arms compared to their wild-type littermates. Similarly, the *Mbnl1^−/−^* mice spent less time in the stretch position which is typically indicative of decreased anxiety (Figure 2B). Lastly, *Mbnl1^−/−^* mice show the same relative number of entries into the closed and open arms, although the total number of entries are decreased (Figure 2C, i, ii and iii, respectively). However, *Mbnl1^−/−^* mice also made fewer head dips over the side of the maze, which normally indicates increased anxiety (Figure 2D).

As with the open field test, the observed behavior may have contributions from decreased motivational drive to explore, muscular phenotypes or altered anxiety. Indeed, *Mbnl1^−/−^* mice have decreased locomotion in the elevated plus maze, making significantly fewer total entries and entries into the closed arms. Although the *Mbnl1^−/−^* mice also entered the open arms fewer times than their wild-type littermates, this was not statistically significant, probably due to the low number of entries by both the wild-type and *Mbnl1^−/−^* groups of mice (Figure 2C, iii). Overall, these results indicate that the musculoskeletal deficits in the *Mbnl1^−/−^* mice lead to poor performance on the elevated plus maze, and result in conflicting indicators of anxiety, namely fewer head dips and less time in the stretch position. Given these results, it is likely that their musculoskeletal phenotype is contributing to poor performance on the elevated plus maze and thus cannot be directly interpreted. Taken together with the Open Field phenotype, these results are inconclusive as to whether or not the *Mbnl1^−/−^* mice experience increased anxiety and but do indicate that the mutant mice have musculoskeletal deficits.

### *Mbnl1^−/−^*, *Dmpk^−/−^* and *Six5^−/−^* mice show normal hippocampus-dependent learning in contextual fear conditioning

To investigate hippocampal cognitive function, we tested these three DM1 models in contextual fear conditioning. This is a hippocampus-dependent task that measures formation of predictive associations. In context fear conditioning, mice are placed in a training chamber, allowed to explore and subsequently given a mild foot shock. In this paradigm, the mouse learns that the specific context predicts the foot shock and will exhibit fear responses such as freezing (lack of all movement except for breathing) when returned to the same context.

**Figure 3:**
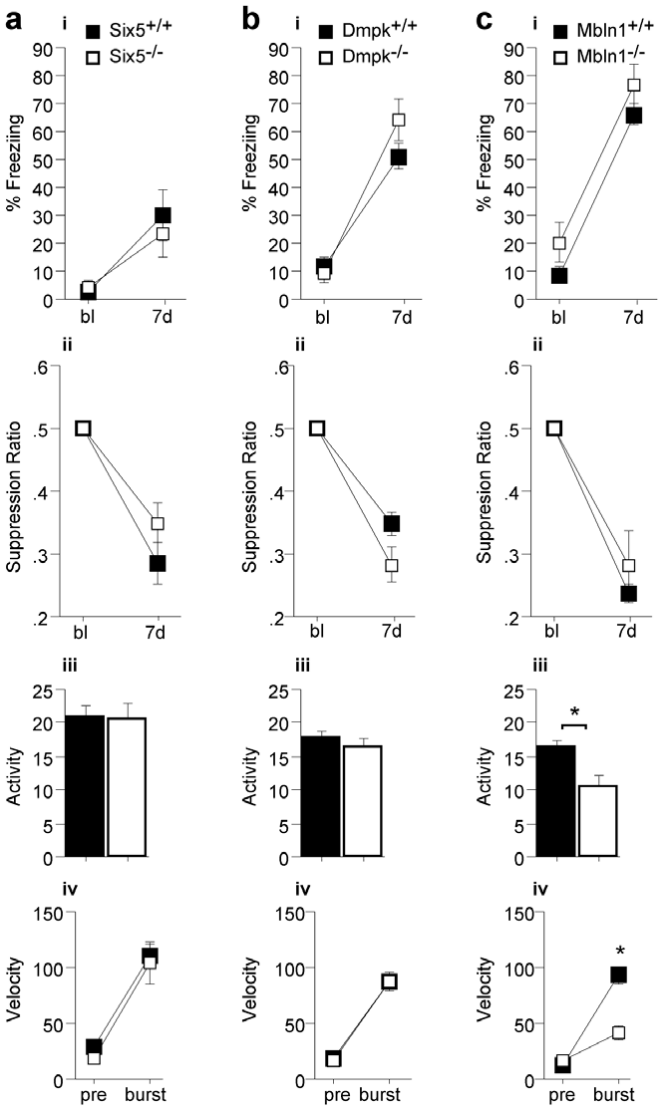
Inactivation of *Six5*, *Dmpk* or *Mbnl1* does not affect Pavlovian contextual fear conditioning. The percent time freezing (panels i) and the activity suppression ratios (a ratio of locomotor activity levels which corrects for hyper- and hypo-activity, panels ii) are shown for baseline (bl) and 7-day (7 d) memory of context fear conditioning. Baseline activity levels (arbitrary units) prior to the foot shock (panels iii) and activity bursts (velocity, cm/s, panels iv) are shown. A) *Six5^−/−^* (white, n = 7) mutant mice display no differences in context fear conditioning freezing, activity suppression, activity level or activity burst phenotypes compared to the *Six5^+/+^* wild type littermate controls (black, n = 6). B) *Dmpk^−/−^* (white, n = 9) mutant mice display no differences in context fear conditioning freezing, activity suppression, activity level or activity burst phenotypes compared to the *Dmpk^+/+^* wild-type littermate controls (black, n = 9). C) *Mbnl1^−/−^* (white, n = ) mutant mice display no context fear conditioning phenotype compared to their *Mbnl1^+/+^* wild-type littermate controls (black, panels i and ii). However, *Mbnl1^−/−^* (white) mutant mice show decreased baseline activity compared to their *Mbnl1^+/+^* wild type controls (black, panel iii) (F(1,14) = 6.86, p = 0.020). *Mbnl1^−/−^* (white, n = 5) mice also display a deficit in their unconditioned response to foot shock (activity burst) compared to their wild type controls (black, n = 12, panel iv) (effect of activity burst x genotype, repeated measures ANOVA F(1,14) = 15, p = 0.0018).

*Six5^−/−^, Dmpk^−/−^* and *Mbnl1^−/−^* mice show no deficits in hippocampus-dependent context fear conditioning (Figure 3Ai, Bi and Ci, respectively). Both freezing (Figure 3A) and activity suppression (the relative decrease in locomotor activity of post-training to pre-training levels, Figure 3B) in a test performed 7 days after training were normal compared to wild-type littermate controls. Activity suppression normalizes for general activity levels of the mice and therefore corrects for potential hypo- and hyper-activity [26].

Although hippocampus-dependent associative conditioning was normal in all three DM1 disease models, abnormal baseline activity and activity bursts (the response to the foot shock which includes jumping and/or running) were observed in *Mbnl1^−/−^* mice. Specifically, both *Six5^−/−^* and *Dmpk^−/−^* mice displayed normal baseline activity (pre-traininig locomotor activity, Figure 3Aiii and Biii, respectively) and foot shock response (Figure 3Aiv and Biv). In contrast, the *Mbnl1^−/−^* mice display hypo-activity (Figure 3Ciii) and a decreased response to foot shock (activity burst, Figure 3Diii), consistent with muscle weakness or myotonia.

### All three mutant strains show normal cognitive function in the Morris water maze, *Mbnl1^−/−^* mice exhibit signs of decreased motivation

To further examine hippocampal function as it relates to visuo-spatial learning and memory, we performed the Morris water maze task. In this task, mice are trained daily to find a hidden platform in a circular pool, using external spatial cues to create a map of the environment and assist in navigation. The Water Maze has non-spatial components such as swim velocity, latency or pathlength during acquisition and thigmotaxis as well as spatial components, such as spatially-selective search strategies during probe trials in which the hidden platform is removed [27]. Mice were given two training trials per day, with probe trials given both during acquisition and at the end of training.

**Figure 4:**
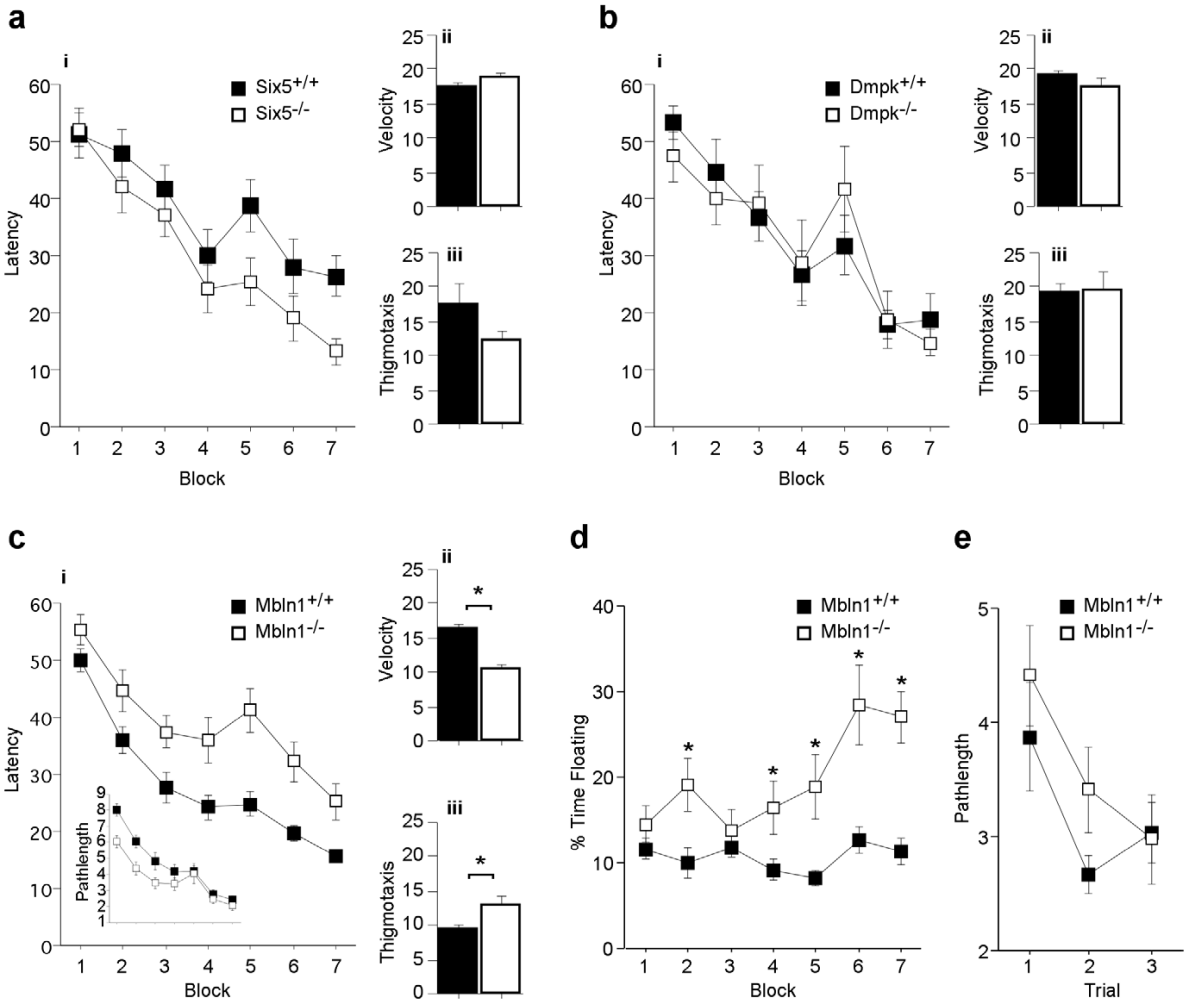
*Mbnl1^−/−^* mutant mice have altered acquisition in the Morris Water Maze. A) No differences were found in the latency (seconds) to find the platform during training (panel i), average velocity (cm/s) during all training days (panel ii) and thigmotaxis (F(1,23) = 2.8, p = 0.11) (% time, panel iii) for *Six5^−/−^* mice (white, n = 7) compared to their wild-type littermate controls, Six5^+/+^ mice (black, n = 6). B) No differences were found in the latency (seconds) to find the platform during training (panel i), average velocity (cm/s) during all training days (panel ii) and thigmotaxis (% time, panel iii) for *Dmpk^−/−^* mice (white, n = 9) compared to their wild-type littermate controls, *Dmpk^+/+^* mice (black, n = 9). C) No statistical differences were found in the latency (seconds) to find the platform during training (panel i) (effect of genotype x latency using 2-day blocks, repeated measures ANOVA, F(6,210) = 1.19, p = 0.31) for *Mbnl1^−/−^* mice (white, n = 13) compared to their wild-type littermate controls, *Mbnl1^+/+^* mice (black, n = 24). C inset: The pathlength (m) to reach the target platform of *Mbnl1^−/−^* (white) was the same as *Mbnl1^+/+^* mice (black) during acquisition (effect of genotype x latency F(6,210) = 1.92, p = 0.080). In contrast, *Mbnl1^−/−^* mutant mice (white) had a slower average velocity during all training days (cm/s, panel ii) (F(1,35) = 38.5, p = <0.0001) and increased thigmotaxis (% time, panel iii) (F(1,35) = 6.47, p = 0.015) compared to their wild-type littermate controls, *Mbnl1^+/+^* mice (black). D) The % time spent swimming slowly (floating behavior) of *Mbnl1^−/−^* (white) increased compared to *Mbnl1^+/+^* mice (black) during acquisition (F(1,35) = 15.7, p = 0.0004; effect of genotype x latency F(6,210) = 6.53, p = <0.0001). *Mbnl1^−/−^* mice showed increased floating behavior in all training blocks except blocks 1 and 3. E) *Mbnl1^−/−^* mice (white) displayed normal acquisition in the visible water maze task (pathlength (m), (effect of genotype x pathlength using 2-day blocks, repeated measures ANOVA, F(6,70) = 0.885, p = 0.42)). Training blocks represent 1 day (2 training trials) for both *Six5^−/−^* and *Dmpk^−/−^* mutant mice, and two days (4 training trials) for *Mbnl1^−/−^* mutant mice. *Mbnl1^−/−^* and *Mbnl^+/+^* mice exhibiting >10% thigmotaxis on the last day of training were excluded from these analyses. See Figure S1 for data including all *Mbnl1* mice.

Both the *Six5^−/−^* (Figure 4A) and *Dmpk^−/−^* (Figure 4B) mutant strains showed normal acquisition as defined by the latency to find the platform (Figure 4i), had normal swim speeds (Figure 4ii), and normal levels of thigmotaxis (Figure 4iii). *Mbnl1^−/−^* mice also improved with time, acquiring the task whether measured as latency (Figure 4Ci) or pathlength to find the platform (Figure 4Ci inset). However, we observed several deficits in the *Mbnl1^−/−^* mice, including decreased swim speeds and increased levels of thigmotaxis (Figure 4Cii and iii, respectively). A number of *Mbnl1^−/−^* mice exhibited >10% thigmotaxis by the end of training so were excluded from these analyses (Figure 4C and D). If these mice are included, the *Mbnl1^−/−^* mice show the same swim speed but even greater thigmotaxis (Figure S1A). These data are consistent with decreased muscle strength observed in the *Mbnl1^−/−^* mice [15].

Interestingly, as training proceeded, *Mbnl1^−/−^* mice spent more time “floating” (% time floating, Figure 4D). This represents an inability to switch from a passive strategy to a spatially selective strategy and is typically indicative of decreased motivation [27]. This phenotype was not observed in either the *Six5^−/−^* or *Dmpk^−/−^* mice (data not shown). This is unlikely to be caused by muscular fatigue or myotonia for the following reasons. First, the *Mbnl1^−/−^* mice perform normally in the visible watermaze (Figure 4E) which involved three sequential trials compared to two sequential trials in the hidden platform task, and was performed after the hidden watermaze. Consequently, if the *Mbnl1^−/−^* mice were fatigued, this should be more evident in the visible watermaze. Second, myotonia exhibits a “practice effect” meaning that the failure of muscle relaxation after contraction decreases with even a short warm-up period of activity [28]. Importantly, this phenotype was not observed at the beginning of training but only manifested after probe trials in which the hidden platform is removed. Thus, these factors are consistent with the hypothesis that the *Mbnl1^−/−^* mice “give up” when the task is too difficult, suggesting motivational deficits instead of muscular fatigue or weakness.

**Figure 5:**
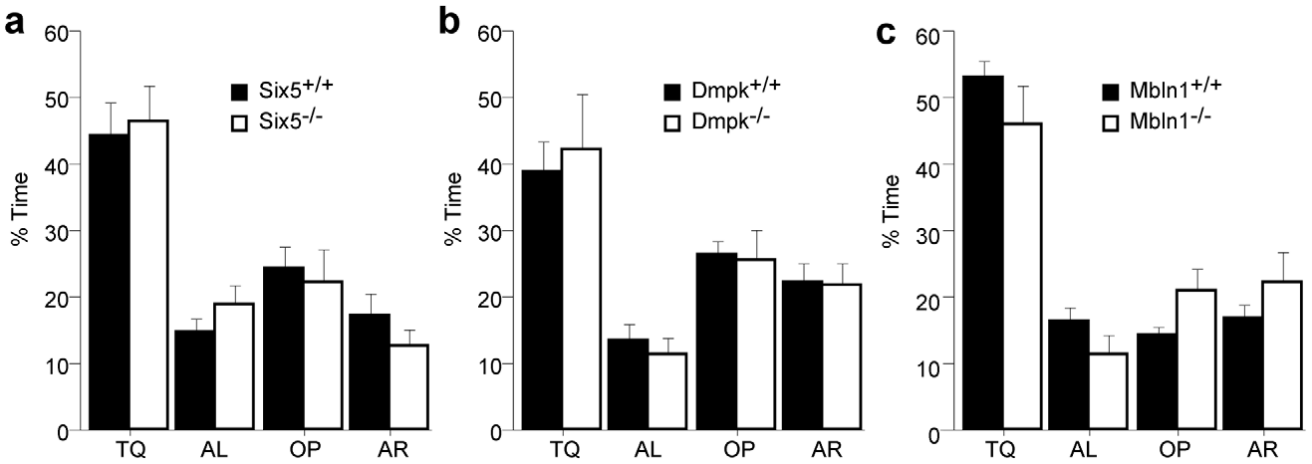
All three mutant mouse strains have normal spatial memory in the Morris Water Maze. The percent time spent in the Target Quadrant (TQ), Adjacent Left (AL), Adjacent Right (AR) or Opposite Quadrant (OP) is shown. A score of 25% reflects random searching. Probe test results after training are shown. A) No differences were found in the percent time spent in the searching in each quadrant on day 7 as shown for *Six5^+/+^* (black, n = 6) and *Six5^−/−^* (white, n = 7) mice. B) The percent time spent searching in each quadrant on day 7 is shown for *Dmpk^+/+^* (black, n = 9) and *Dmpk^−/−^* (white, n = 9) mice. C) No differences were found in the percent time spent in searching in each quadrant on day 13 as shown for *Mbnl1^+/+^* (black, n = 24) and *Mbnl1^−/−^* (white, n = 13) mice (genotype x quadrant, repeated measures ANOVA F(3,105) = 2.20, p = 0.092).

*Six5^−/−^*, *Dmpk^−/−^* and *Mbnl1^−/−^* mutant mice showed normal spatial learning and memory, searching selectively for the platform (Figure 5A, B, and C respectively). If, however, spatial learning and memory is assessed with the inclusion of the *Mbnl1^−/−^* mutant mice that exhibited high levels of thigmotaxis at the end of training, they showed a deficit in spatial memory, randomly searching for the platform in all quadrants (% time in target quadrant Figure S1D). Importantly, *Mbnl1^−/−^* mutant mice had normal acquisition when trained in the visible water maze task which is marked by a local cue and has the same physical requirements but involves different cognitive capacities (Figure 5E and Figure S1C). Therefore, the visible water maze controls for performance indicating that the decrease spatial ability in the *Mbnl1^−/−^* mice is not due to decreased vision from cataracts, or muscular complications such as myotonia, decreased skeletal muscle strength or cardiac complications.

### *Mbnl1^−/−^* mice show decreased sucrose consumption in a test for anhedonia

**Figure 6:**
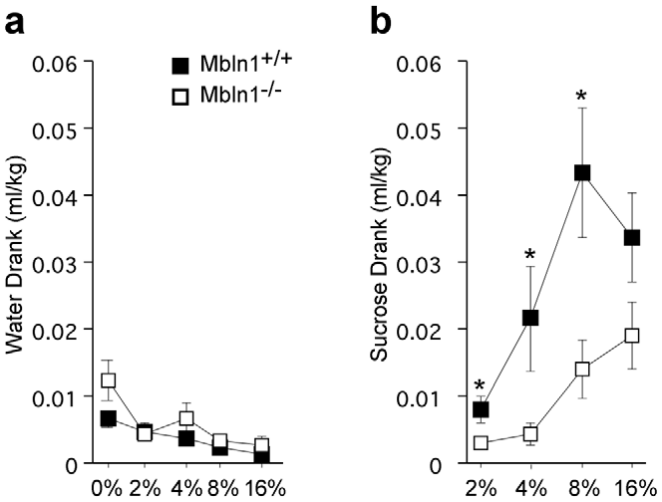
Inactivation of *Mbnl1* results in decreased sucrose consumption, a measure of motivation. A) *Mbnl1^−/−^* (white, n = 14) mutant mice consume the same amount of water during habituation (0%) as well as during presentation of sucrose (2, 4, 8 and 16%) (genotype x water, repeated measures ANOVA F(4,104) = 1.67, p = 0.16) main effect of genotype (F(1,26) = 2.64, p = 0.12) compared to their wild-type littermate controls (black, n = 14). B) In contrast, *Mbnl1^−/−^* mutant mice consume less sucrose, regardless of the percent sucrose (genotype x sucrose, repeated measures ANOVA F(3,78) = 3.79, p = 0.014) main effect of genotype (F(1,26) = 6.23, p = 0.018). The *Mbnl1^−/−^* mutant mice drink less 2% (ANOVA F(1,26) = 4.96, p = 0.035), 4% (ANOVA F(1,26) = 4.46, p = 0.044), and 8% (ANOVA F(1,26) = 7.71, p = 0.010) sucrose but statistically consume the same amount of 16% (ANOVA F(1,26) = 3.00, p = 0.095) sucrose as their wild-type littermates, indicating that the mutant mice can taste and discriminate between water and the sweet solution.

The performance of the *Mbnl1^−/−^* mice in the watermaze suggested that these mutants may have motivational deficits. To directly test apathy in these mutants, we chose a task that has minimal physical requirements. The sucrose consumption test, a classic anhedonia test, presents the mouse with a two bottle choice, one bottle contains water and the other contains a solution of sucrose. During habituation, *Mbnl1^−/−^* mice drank the same amount of water as their wild-type controls (Figure 6A). However, *Mbnl1^−/−^* mice showed no preference for sucrose compared to the wild-type mice for 2%, 4% and 8% sucrose (Figure 6B). Given a large enough reward of 16% sucrose, *Mbnl1^−/−^* mice showed a increased consumption of sucrose, drinking a similar volume of sucrose as their wild-type littermates (Figure 6B). The *Mbnl1^−/−^* mice showed a lack of interest in sucrose, a behavior typical of decreased motivation.

## Discussion

Patients with myotonic dystrophy have numerous physical symptoms, including musclular, skeletal, cardiac and vision difficulties. It has been long realized that they experience cognitive impairment, with a lower IQ overall and specific deficits in attention, visuo-spatial function, perception, executive function and autism spectrum disorder symptoms [16] [29] [17] [19] [20]. Apathy, hypersomnia and anxiety have been more recently associated with myotonic dystrophy and are dissociable from the physical disabilities. They appear to be explicit symptoms of the disease, and like the physical symptoms, there is a correlation with the severity of disease and cognitive deficits in which less severely affected patients generally have milder cognitive deficits.

The pleiotropy of this disease is reflected in symptoms, their severity and the molecular alterations that occurs. The disease is caused by a trinucleotide repeat expansion situated in the 3′UTR of the *DMPK* gene, located immediately 5′ of the *SIX5* gene but the core of the biochemical defect is sequestration of MBNL1 by the expanded trinucleotide repeat RNA in nuclear foci. RNA processing is disrupted, in part due to inactivation of the alternative splice regulator, MBNL1 which normally regulates splicing of several transcripts including *Cncl1*, a chloride channel associated with myotonia [30] [15]. This RNA-dominant disease consequently has many possible molecular alterations that lead to individual symptoms. Therefore, it is important to ascertain which molecular alteration leads to the cognitive and emotional deficits seen in DM1 patients.

Neither *Six5^−/−^* nor *Dmpk^−/−^* mice appear to affect cognitive function in mouse models of DM1. In DM1, a trinucleotide repeat is expanded and disrupts normal RNA processing in the nucleus, and sequestering Mbnl1 protein in specific foci. Since in both *Six5^−/−^* and *Dmpk^−/−^* mice, there is no trinucleotide repeat, these mutants can be used to differentiate between the direct effect of a gene and the effect of impairing the function of other proteins with which they interact. Supporting the hypothesis that RNA metabolic disruption is key to the development of DM1 symptoms, we found deficits only in the *Mbnl1^−/−^* mice. *Mbnl1^−/−^* mice have normal spatial learning and memory but show severe alterations in motivated behavior. Specifically, *Mbnl1^−/−^* mice showed signs of motivation and apathy in a physically demanding task (Morris water maze) or a simple task (sucrose consumption).

Due to their physical symptoms, the cognitive and emotional phenotype of the *Mbnl1^−/−^* mice is difficult to assess. However, *Mbnl1^−/−^* mice show unmotivated behavior in the water maze, failing to switch from passive strategies to a spatially selective search as exhibited by increased floating in the watermaze task [27]. Interestingly, this increased floating only manifested after probe trials were performed. Combining this observation with normal performance in the visual watermaze suggests a complex interaction between the difficulty of the task and a substantial motivational component that underlie their lack of spatial acquisition, with the possibility that muscular weakness also contributes to apathy. Thus, *Mbnl1^−/−^* mice may become unmotivated if they fail to learn the platform location expediently. A number of *Mbnl1^−/−^* mice also exhibited such pronounced thigmotaxis that they were excluded from final analysis. The remaining *Mbnl1^−/−^* mice showed normal spatial learning and memory when tested without the platform present, however, the *Mbnl1^−/−^* mice that demonstrated pronounced thigmotaxis did not show any spatial learning and memory. This demonstrates that Mbnl1 deficit in these mice results in a range of severity of the observed phenotypes.

To substantiate a phenotype of decreased motivation, we tested *Mbnl1^−/−^* mice for apathy towards sucrose. The mice were tested in their home cage with the sucrose and water bottles presented in their normal location. Therefore, physical complications of this task are minimal as the physical demands are the same as drinking water in their home cage: these mutants do not experience dehydration from an inability to reach the water bottle. In this test for anhedonia, *Mbnl1^−/−^* mice show preference for sucrose over water at a much reduced rate than their wild-type counterparts. The *Mbnl1^−/−^* mice appear to require a higher reward as a 16% solution was equally appealing to the *Mbnl1^−/−^* mice as a 4% solution was to the wild-type mice. This suggests that the mutant mice do not have an innate aversion to sucrose but instead that they have a decreased interest in seeking a sweet reward. This finding may be particularly relevant as apathy is a striking feature in myotonic dystrophy patients and has been shown to be independent of both clinical depression and peripheral muscular weakness [17].

In addition to lack of motivation, myotonic dystrophy patients have increased anxiety. We tested all three mutant mouse strains for alterations in anxiety levels and only *Mbnl1^−/−^* mice exhibited high levels of thigmotaxis, a behavior indicative of anxiety. Although *Dmpk^−/−^* and *Six5^−/−^* showed no overt anxiety phenotype in the open field, we performed only one test of anxiety and thus subtle phenotypes may exist that can be detected using other tests. In a second test of anxiety, the elevated plus maze, the *Mbnl1^−/−^* mice showed conflicting signs of increased and decreased anxiety. Given these ambiguous results, it is difficult to determine if these mice are good models of anxiety observed in myotonic dystrophy patients. Furthermore, it would be difficult to determine the basis for their altered performance in either the open field and elevated plus maze tasks. The underlying cause of altered performance could be decreased motivation to explore, increased anxiety or musculoskeletal effect, or a combination of all three factors, all three of which are consistent with the human disease.

Finally, contextual associative learning was normal in the *Mbnl1^−/−^* mice. Since DM patients do not show remote memory deficits, we did not test memory at later times. Specifical alterations in 24-hour memory consolidated and remote memory were not tested however, based on a normal memory phenotype at seven days, which is it is indicative of protein-synthesis dependent memory and the transition to a remote memory [31], [32], we speculate that all three mutant strains will show relatively normal memory at these times as well. Taken together, these data suggest a normal spatial learning, with a complex motivational alteration that may result in part through an interaction of muscular effects with emotional state in *Mbnl1^−/−^* mice.

Important questions to address in the future will be to assess motivational drive in *Mbnl1^−/−^* mice with normal musculoskeletal function. Since these are separable in patients with DM1, it is likely that the transcripts controlled by Mbnl1 processing may play a specific role in apathy and motivation. The specific transcripts that undergo altered splicing due to Mbnl1 are not all known. Deep sequencing of the different mutant models of DM1 will be very useful in generating and further refining genotype/phenotype relationships. Mbnl1 belongs to a three-member family, and the role of specific Mbnl family members may further refine our understanding of the transcriptional misregulation that leads to specific symptoms [33]. The lack of these specific phenotypes in the *Dmpk^−/−^* and *Six5^−/−^* mice suggests that these genes do not play a role in motivation when they are specifically deleted but their dysfunction, as caused by the DM1 trinucleotide repeat expansion, may still participate in generating cognitive effects in patients. Defining the specific regions of the brain that show functional impairment in Mbln1 knockout mice and identification of Mbnl1 target RNAs in these areas will be critical to understanding this complex phenotype. These lines of inquiry should allow key insights into the molecular targets and circuits that regulate motivation, and spatial learning and memory in humans.

## Materials and Methods

### Mice

All experiments were performed in accordance with the institutional guidelines of the University of California at Los Angeles. For all tasks, male and female mice in the 129B6 genetic background were generated from heterozygous matings and all tests used mutant mice with their respective wild type littermates as controls. All mice were group-housed, maintained in a 12∶12 light/dark cycle and had food and water ad libitum. *Six*5*^−/−^*, *Dmpk^−/−^*, and *Mbnl1^−/−^* mice have been previously described [15], [22], [23], [24], [25]. All mice were tested between the ages of 8–14 months, *Six5^−/−^* mice ranged from 8–12 months, *Dmpk^−/−^* mice were 8–10 months and *Mbnl1^−/−^* mice were 8–14 months at the time of testing. Mice were used for multiple tests in this order: open field, watermaze, fear conditioning or open field, elevated plus maze, sucrose consumption, watermaze with a minimum of two weeks rest before commencing a new test.

### Behavioral Experiments

Since physical deficits are present, we looked at specific measures during the behavioral tasks to minimize the influence of these impairments.

Open Field activity, the first behavioral test performed, was assessed to examine generalized activity and thigmotaxis (wall-hugging). Animals were placed in an open 30.5 cm by 30.5 cm space and allowed to explore for ten minutes under dim light conditions. Horizontal locomotor acivity was measured as beam breaks by paired sets of photo beams using the Activity Monitor systems (Med Associates, St. Albans, Vt). We measured thigmotaxis (amount of time in the outer versus inner zone), and general activity levels (velocity and total distance travelled). For the *Six5* mutant strain, 6 wild-type and 7 mutant mice were tested. For the *Dmpk* mutant strain, 9 wild-type and 9 mutant mice were tested. For the *Mbnl1* mutant strain, 23 wild-type and 19 mutant mice were tested.

In the elevated plus maze, a second anxiety test, mice were placed in the center of a four-arm maze and their movement on the maze was recorded for five minutes in dim light. The maze was 60 cm off the floor. Two arms were sheltered with 16.5 cm opaque walls and the other two arms were open. We measured the amount of time in the closed and open arms and the center of the maze, as well as the number of entries into each arm. The time in a stretch approach position and the number of times the mice dipped their heads over the edge of the maze was also scored. We tested 14 wild-type and 14 *Mbnl1^−/−^* mice.

Mice were trained in context fear conditioning. Animals were placed in the training chamber during which time baseline activity and freezing levels were obtained, followed by a single shock, delivered at 4 minutes. After seven days, mice were returned to the training chamber and tested for long-term memory of the context by measuring activity and freezing levels. Context fear conditioning was performed as previously described [31] using Med Associated Mouse Context Conditioning boxes. Automated freezing, activity scores and activity suppression ratios [(test activity)/(test activity + baseline activity)] were calculated as previously described [26]. Six wild-type and 6 Six5*^−/−^* mice, 10 wild-type and 6 Dmpk*^−/−^* mice, and 12 wild-type and 5 *Mbnl1^−/−^* mice were tested in this task.

Morris Water Maze training was commenced to examine spatial learning and memory of the mutant mice, as previously described [34]. Briefly, animals are trained to locate a hidden submerged platform in a pool filled with opaque water. Probe trials, used to assess spatial memory by examining the search pattern of the mice when the platform was removed, were administered on days 5, 7, 9 for *Dmpk^−/−^*, days 4 and 7 for *Six5^−/−^* and days 4, 7, 10 and 13 for *Mbnl1^−/−^* mice. Results for the last probe trial are shown. Pathlengths to find the hidden platform during acquisition were used in addition to latency as latencies can be influenced by slow swim speeds. Upon completion of the hidden Water Maze task, all mice were tested in the visible water maze using a single session with three training trials in which the location of the submerged platform is marked by a local cue. This test further controls for performance, including visual capabilities and muscle deficits. *Mbnl1^−/−^* mice were analysed in two groups, data shown excludes individual mice that showed >10% thigmotaxis at the end of training as these mice may have visual complications that affect distant vision. Analysis of all *Mbnl1^−/−^*mice is shown in the supplemental data. For the *Dmpk* mutant strain, we tested 10 wild- type and 6 mutant mice, which were trained with two trials per day. For the *Six5* mutant strain, we tested 13 wild-type and 12 mutant mice with two trials per day with probe trials on days 4 and 7. For the *Mbnl1* mutant strain, we tested 25 wild type and 21 mutant mice with two trials per day with probe trials. Analysis of *Mbnl1^−/−^* mice was performed both excluding and including mice that exhibited more than 10% thigmotaxis at the end of training. This resulted in exclusion of 1 wild type and 8 *Mbnl1^−/−^* mice.

Mice were tested for motivation/anhedonia using the sucrose consumption test. Animals were water restricted overnight on each day of habituation and testing. Mice were habituated to testing cages with two drinking spouts, both of which contained water after which, one solution was replaced with sucrose. Mice were given 2%, 4%, 8% and 16% sucrose for three days each, the first day was used as habituation to the new solution. The volume of either water or sucrose was averaged for the remaining two days, in which the presentation of water and sucrose was counterbalanced. Fourteen wild-type and 14 *Mbnl1^−/−^* mice were tested.

### Data Analysis

The following software was used for analysis of raw data: Open Activity from Med Associates for the Open Field, custom designed software for fear conditioning described [26] and HVS Image for the water maze. All other behavioral data was obtained by hand-scoring. ANOVA was used to compare genotypes in the Open Field (thigmotaxis, velocity and ambulation), Water Maze (thigmotaxis and velocity), and Fear Conditioning (% freezing and suppression ratio for baseline and 7 day test, and activity). Repeated Measures ANOVA was used to compare genotypes in the Water Maze (acquisition latency or pathlength, % floating, visible Water Maze latency, and % time in target quadrant), and Fear Conditioning (activity bursts). All error bars represent standard error of the mean.

## Supporting Information

Figure S1 Mbnl1−/− mutant mice have altered performance in the Morris Water Maze. All mice, including Mbnl1−/− mice that demonstrate pronounced thigmotaxis are included. A) No statistical differences were found in the latency (seconds) to find the platform during training (panel i) (effect of genotype x latency using 2-day blocks, repeated measures ANOVA, F(6,264) = 1.29, p = 0.26) for Mbnl1−/− mice (white, n = 21) compared to their Mbnl1+/+ wild-type littermate controls (black, n = 25). A inset: The pathlength (m) to reach the target platform of Mbnl1−/− (white) was significantly different during acquisition compared to Mbnl1+/+ mice (black) (effect of genotype x latency F(6,264) = 2.81, p = 0.012; main effect of genotype F(1.44) = 7.43, p = 0.0092). Mbnl1−/− mutant mice (white) had a slower average velocity during all training days (cm/s, panel ii) (F(1,43) = 52.7, p = <0.0001) and increased thigmotaxis (% time, panel iii) (F(1,43) = 12.2, p = 0.0011) compared to their wild-type littermate controls, Mbnl+/+ mice (black). B) The % time spent swimming slowly (floating behavior) of Mbnl1−/− (white) increased compared to Mbnl1+/+ mice (black) during acquisition (F(1,44) = 16.4, p = 0.0002; effect of genotype x latency F(6,264) = 5.25, p = <0.0001) (panel i). Mbnl1−/− mice showed increased floating behavior in all training blocks except block 3. C) Mbnl1−/− mice displayed normal acquisition in the visible water maze task (pathlength (m), (effect of genotype x pathlength using 2-day blocks, repeated measures ANOVA, F(2,86) = 0.694, p = 0.50)). Training blocks represent two days (4 training trials) for Mbnl1 mutant mice. D) The percent time spent in the Target Quadrant (TQ), Adjacent Left (AL), Adjacent Right (AR) or Opposite Quadrant (OP) is shown. A score of 25% reflects random searching. Probe test results after training are shown. Statistically significant differences were found in the percent time spent in searching in each quadrant on day 13 as shown for wild-type (black) and Mbnl1−/− (white) mice (genotype x quadrant, repeated measures ANOVA F(3,132) = 5.66, p = 0.0011), main effect of genotype (F(1,44) = 0.041, p = 0.84). The Mbnl1−/− mice spent significantly less time searching in the target quadrant (F(1,44) = 7.48, p = 0.0089) and more time in the opposite quadrant (F = (1,44) = 8.84,p = 0.0048).
(0.17 MB TIF)
