# Supplementary figures and images for: Muscleblind1, but Not Dmpk or Six5, Contributes to a Complex Phenotype of Muscular and Motivational Deficits in Mouse Models of Myotonic Dystrophy
Source: PLoS One. 2010 Mar 25;5(3):e9857. doi: 10.1371/journal.pone.0009857 (PMC2845609; doi:10.1371/journal.pone.0009857)

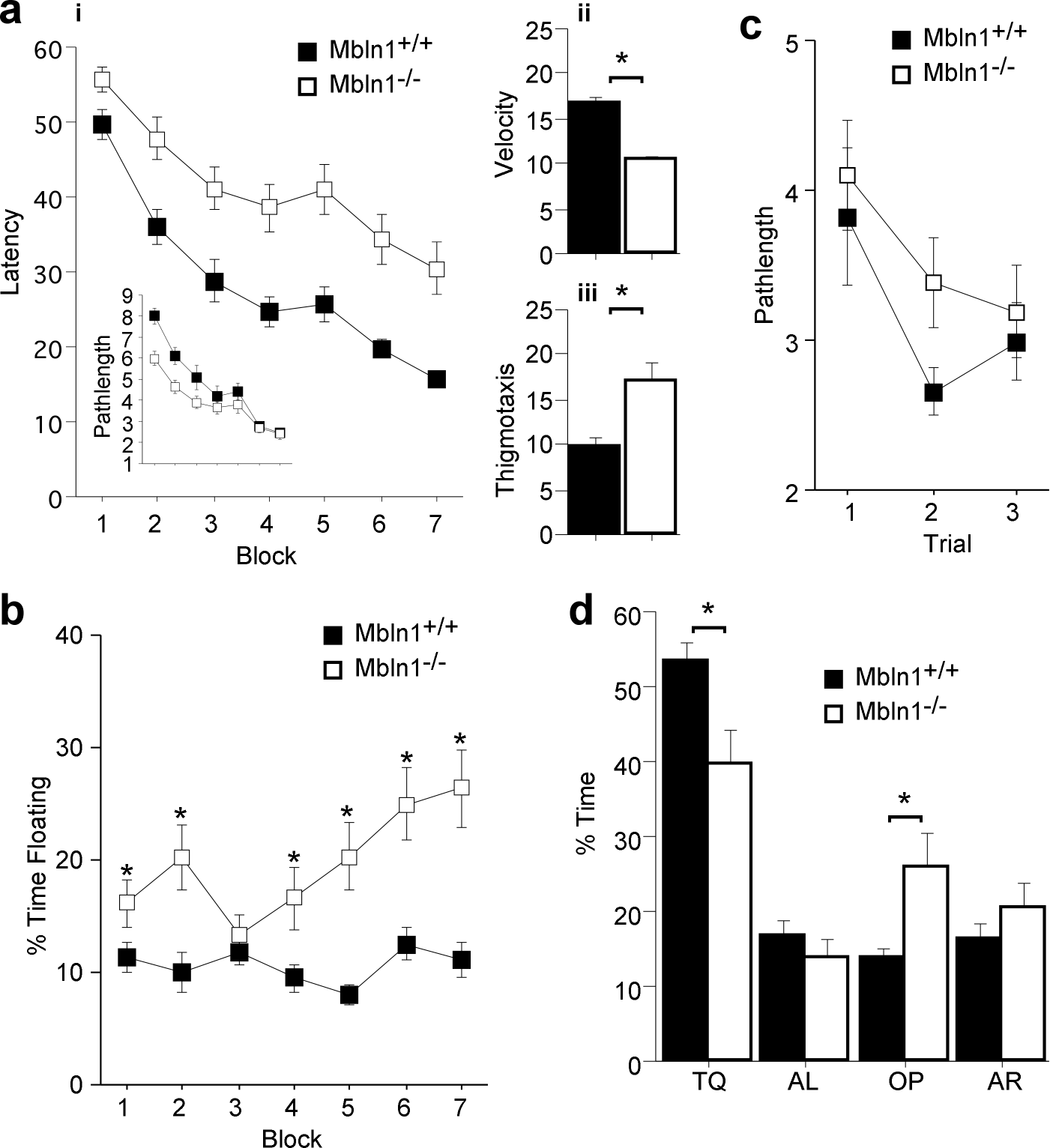

Supplement: Figure S1 — Mbnl1−/− mutant mice have altered performance in the Morris Water Maze. All mice, including Mbnl1−/− mice that demonstrate pronounced thigmotaxis are included. A) No statistical differences were found in the latency (seconds) to find the platform during training (panel i) (effect of genotype x latency using 2-day blocks, repeated measures ANOVA, F(6,264) = 1.29, p = 0.26) for Mbnl1−/− mice (white, n = 21) compared to their Mbnl1+/+ wild-type littermate controls (black, n = 25). A inset: The pathlength (m) to reach the target platform of Mbnl1−/− (white) was significantly different during acquisition compared to Mbnl1+/+ mice (black) (effect of genotype x latency F(6,264) = 2.81, p = 0.012; main effect of genotype F(1.44) = 7.43, p = 0.0092). Mbnl1−/− mutant mice (white) had a slower average velocity during all training days (cm/s, panel ii) (F(1,43) = 52.7, p = <0.0001) and increased thigmotaxis (% time, panel iii) (F(1,43) = 12.2, p = 0.0011) compared to their wild-type littermate controls, Mbnl+/+ mice (black). B) The % time spent swimming slowly (floating behavior) of Mbnl1−/− (white) increased compared to Mbnl1+/+ mice (black) during acquisition (F(1,44) = 16.4, p = 0.0002; effect of genotype x latency F(6,264) = 5.25, p = <0.0001) (panel i). Mbnl1−/− mice showed increased floating behavior in all training blocks except block 3. C) Mbnl1−/− mice displayed normal acquisition in the visible water maze task (pathlength (m), (effect of genotype x pathlength using 2-day blocks, repeated measures ANOVA, F(2,86) = 0.694, p = 0.50)). Training blocks represent two days (4 training trials) for Mbnl1 mutant mice. D) The percent time spent in the Target Quadrant (TQ), Adjacent Left (AL), Adjacent Right (AR) or Opposite Quadrant (OP) is shown. A score of 25% reflects random searching. Probe test results after training are shown. Statistically significant differences were found in the percent time spent in searching in each quadrant on day 13 as shown for wild-type (black) an [file pone.0009857.s001.tif]
